# A Case of p63 Positive Diffuse Large B Cell Lymphoma of the Bladder

**DOI:** 10.1155/2016/4348208

**Published:** 2016-08-25

**Authors:** Chelsey D. Deel, Carol Jones, Teresa Scordino

**Affiliations:** ^1^Department of Pathology, University of Oklahoma Health Sciences Center, P.O. Box 26901, BMSB 451, Oklahoma City, OK 73126, USA; ^2^Ventana Medical Systems, 1910 E. Innovation Park Dr, Tucson, AZ 85755, USA

## Abstract

Diffuse large B cell lymphoma (DLBCL), currently the most common type of non-Hodgkin lymphoma (NHL), is an aggressive B cell neoplasm that typically presents in older adults as a rapidly enlarging mass. The enlarging mass typically represents a lymph node, although extranodal disease can occur in a significant percentage (40%) of cases. The most common extranodal sites of involvement include the gastrointestinal tract and skin; primary bladder lymphoma represents only 0.2% of extranodal non-Hodgkin lymphomas. We report a case of diffuse large B cell lymphoma occurring in the bladder of an 83-year-old gentleman with an initial presentation of hematuria. This neoplasm displayed large, atypical cells with vesicular chromatin and prominent nucleoli that involved the bladder mucosa with invasion into muscularis propria, prostate, and urethra. Positive staining for p63 initially raised suspicion for poorly differentiated urothelial carcinoma; however, lack of staining for pancytokeratin and positive staining for LCA, CD20, CD79a, and PAX-5 confirmed the diagnosis of diffuse large B cell lymphoma. Though it does not occur in all cases, p63 can be positive in a significant percentage of cases of DLBCL; therefore, a diagnosis of lymphoma remains an important entity on the differential diagnosis of aggressive and particularly poorly differentiated neoplasms.

## 1. Introduction

Diffuse large B cell lymphoma (DLBCL) represents the most common type of non-Hodgkin lymphoma (30–40% of NHL cases) [1]. This aggressive B cell neoplasm typically occurs in adults with a median age in the seventh decade. It has slight male predominance. It commonly presents as an enlarging lymph node mass, although extranodal disease is present in up to 40% of cases. The most common sites of extranodal involvement include the gastrointestinal tract and skin; primary lymphoma of the bladder is extremely rare, representing only 0.2% of extranodal NHL and <1% of bladder tumors [2]. Primary bladder lymphomas typically are low-grade neoplasms, such as mucosa-associated lymphoid tissue (MALT) lymphoma; however, 20% of bladder lymphomas are high-grade neoplasms, with the majority being diffuse large B cell lymphoma [2]. In extranodal sites, these tumors cause obliteration of normal epithelial components by large, atypical cells with vesicular chromatin and prominent nucleoli. The immunophenotype of DLBCL is heterogeneous, with a majority of tumors displaying pan B cell markers such as CD19, CD20, and CD22. CD10 expression occurs in 25–50% of cases, with variable expression of BCL2 and BCL6 [1]. Though it is often used as a marker of squamous cell carcinoma, p63 can also be positive in a significant percentage of DLBCL cases [3–7]. Its effect on prognosis and survival continues to be studied; however, studies concur that p63 plays a role in DLBCL tumor progression. In particular, the TAp63 isoform appears to be associated with hematologic malignancy, while the ΔNp63 isoform is more commonly associated with squamous cell carcinoma. This potential phenomenon emphasizes the importance of considering lymphoma on the differential diagnosis of extranodal neoplasms.

## 2. Case Report

We present a case of an 83-year-old gentleman with a past medical history of prostate cancer status after radiation treatment, hypertension, BPH, hyperlipidemia, and renal impairment who presented with gross hematuria. A transurethral resection revealed an infiltrating, poorly differentiated tumor with abundant apoptosis and mitotic activity showing invasion into the muscularis propria. Due to the patient's history of prostate carcinoma, immunohistochemical stains were performed. The malignant cells were positive for p63 and negative for PSA, cytokeratin 7 (CK7), and cytokeratin 20 (CK20); therefore, a diagnosis of poorly differentiated urothelial carcinoma was favored. A CT scan of the abdomen and pelvis was remarkable for a remarkably thickened bladder wall with ill-defined boundaries and dilation of the distal right ureter. Small pelvic lymph nodes were also visualized.

**Figure 1:**
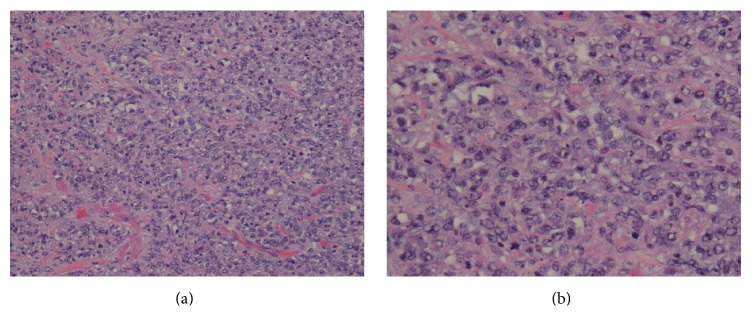
(a) Medium and (b) high power images display a neoplastic proliferation of large, atypical cells with vesicular chromatin and prominent nucleoli. (Hematoxylin-eosin stain; (a) 20x magnification and (b) 40x magnification.)

**Figure 2:**
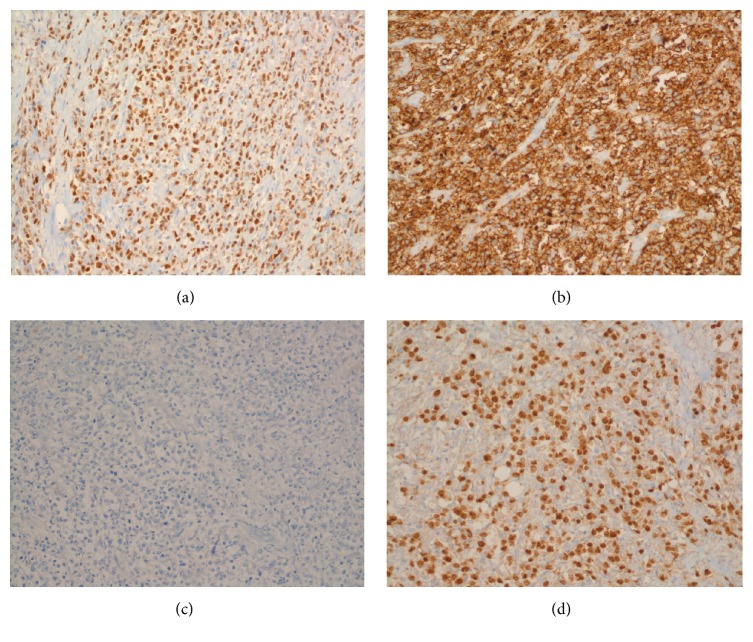
Immunohistochemical staining pattern and diffuse large B cell lymphoma of the bladder. (a) p63 positive, (b) CD20 diffusely positive, (c) pancytokeratin negative, and (d) PAX-5 positive.

Subsequent sampling revealed an aggressive neoplasm with invasion to bladder muscularis propria, prostate, and urethra. The tumor displayed large malignant cells with vesicular chromatin and prominent nucleoli arranged in a mildly cohesive pattern (Figure 1). Based on morphology, a differential diagnosis of poorly differentiated urothelial carcinoma, pleomorphic plasmacytoid variant of urothelial carcinoma, and lymphoma was considered. Immunohistochemical stains were performed. The malignant cells were positive for p63, CD20, LCA, CD79a, and PAX5 and negative for pancytokeratin, CD138, CD30, CD34, CD3, and CD5 (Figure 2). The overall morphologic and immunophenotypic findings were consistent with diffuse large B cell lymphoma. The patient is alive after one and a half years and currently being followed under close observation.

## 3. Discussion

Diffuse large B cell lymphoma may present in extranodal sites. This case illustrates the importance of considering a diagnosis of lymphoma when confronted with a poorly differentiated neoplasm. Expression of p63 by lymphoma cells presents a potential diagnostic pitfall. Review of the literature reveals several reports of p63 expression in high-risk diffuse large B cell lymphoma. P63 is positive in a significant amount of DLBCL cases, with percentages ranging from 15 to 70% depending upon the study examined [3–7]. The correlation between p63 expression and overall survival remains unclear; however, studies have shown a correlation of p63 with a high proliferative index, suggesting its involvement with DLBCL tumor progression [5, 6].

The* p63* gene, homolog of the* p53* tumor suppressor gene, is located on chromosomes 3q27-3q28 and encodes proteins responsible for p53 activation and apoptosis [6, 7]. P63 is consistently expressed in a variety of normal tissues, including squamous epithelia and urothelium, basal bronchial cells of the lung, and basal glandular cells of the breast and prostate. In addition, p63 is expressed in occasional germinal center and peripheral blood lymphocytes [6]. It is known to play a critical role in the survival and differentiation of stratified epithelium and epithelial tumors; however, its role in lymphoid function and the progression of lymphoid malignancy remains unclear.

P63 comprises a group of six different proteins produced through alternative splicing of a single mRNA transcript [8]. These isoforms, among other functions, are responsible for the regulation and differentiation of stratified epithelial cells. Changes in isoform expression lead to proliferation and differentiation of these tissues and also play an active role in neoplasia [5, 8]. While* p53* is widely acknowledged as a tumor suppressor gene and its mutations are responsible for tumor progression, the role of* p63* as a cancer oncogene continues to be studied. Two particular groups of p63 isoforms, TAp63 and ΔNp63, have received further study in the development of neoplasia.

The TAp63 isoform contains an N-terminal transactivation domain with sequence homology to the p53 transactivation domain. This isoform is responsible for transactivating* p53* target genes and inducing apoptosis. The ΔNp63 isoform lacks this N-terminal domain and likely has a dominant-negative effect on p53 [8]. RT-PCR and Western Blot analysis of various cancer cell lines reveal that the ΔNp63 isoform is overexpressed in squamous cell carcinomas of the head, neck, and lung, while overexpression of the TAp63 isoform appears to be responsible for lymphoid malignancy [5, 8]. This implies a tumor-promoting role, though the exact mechanism is not clear.

Recent studies [9, 10] have evaluated the application of immunohistochemical stains specific for the TAp63 and ΔNp63 isoforms in the diagnosis of carcinoma. Currently, the widely used pan-p63 immunohistochemical stain does not discriminate between the various p63 isoforms and can demonstrate cross-reactivity with p53 and p73. These studies suggest usage of stains specific for the TAp63 and ΔNp63 (also known as p40) isoforms will allow better discrimination of carcinoma from various sites. However, there is little published data on the staining pattern of these antibodies in lymphoma; therefore, further studies are warranted to evaluate their usefulness in differentiating poorly differentiated carcinoma from lymphoma. These immunohistochemical stains were not evaluated in our case, as they were not available in our laboratory at the time.

As previously noted, the* p63* gene responsible for creating these various protein isoforms is located on chromosomes 3q27-3q28. Interestingly, the* Bcl-*6 gene is also located on chromosome 3q27, adjacent to the* p63* locus. Given that rearrangements in the* Bcl-6* gene are seen in approximately 30% of diffuse large B cell lymphomas [1], some authors have speculated that the close relationship of* p63* to* Bcl-6* may contribute to its potential involvement in DLBCL tumor progression [6].

Several studies have adopted the task of analyzing p63 expression in diffuse large B cell lymphoma and its prognostic impact, fueled by an earlier study by Di Como et al. that showed high levels of p63 in a subset of DLBCL (25%) and follicular lymphoma (22%) [11]. These studies confirmed significant expression of p63 in diffuse large B cell lymphoma, with percentages ranging from 15 to 70%. No consistent correlation was found between the immunohistochemical expression of p63 and expression of CD10, BCL-2, BCL-6, or p53 [7]. However, studies by Hedvat et al. found a correlation between p63 expression and Ki-67 proliferative index, suggesting a role in DLBCL tumor progression [5, 6]. Despite this finding, investigations into the effect of p63 on prognosis have found conflicting results. Two studies by Park et al. suggest a decreased survival with p63 overexpression in cases of DLBCL [3, 4]; however, another study by Hallack Neto et al. evaluating cases of high-risk diffuse large B cell lymphoma found better disease-free survival in patients with p63-positive tumors than those that were p63-negative [7]. Additional studies reveal no significant correlation with p63 expression and overall survival, further complicating the picture [5, 6]. It remains likely that these studies used different parameters to define survival; therefore, a larger, more comprehensive study is warranted to truly evaluate the role of p63 expression on prognosis in DLBCL.

## 4. Conclusion

This case illustrates the importance of considering hematologic malignancies in the differential diagnosis of poorly differentiated neoplasms and serves as a reminder that no single immunohistochemical marker is entirely specific for a certain tumor type. Incorporation of morphology and immunophenotype is vital to final diagnosis.* P63*, homolog of the tumor suppressor p53, has an important role in the development and differentiation of stratified epithelia; as such, it is usually interpreted as a marker of epithelial differentiation/carcinoma. However, as illustrated by this case, p63 can be positive in a significant subset of cases of diffuse large B cell lymphoma. Of particular note, the isoform TAp63 appears to be involved in the development of lymphoma, while the ΔNp63 isoform is typically seen in cases of squamous cell carcinoma. Numerous studies support this phenomenon and correlate p63 expression with DLBCL tumor progression; its impact on prognosis and survival remains to be seen.
